# Controlled optical near-field growth of individual free-standing well-oriented carbon nanotubes, application for scattering SNOM/AFM probes

**DOI:** 10.1515/nanoph-2022-0378

**Published:** 2022-10-17

**Authors:** Payam Yazdanfar, Hesam Heydarian, Bizhan Rashidian

**Affiliations:** Department of Electrical Engineering, Sharif University of Technology, Tehran, Iran

**Keywords:** CNT-tips, localized heat generation, maskless selective growth, optical near field, self-aligned catalyst, temperature rise gradient

## Abstract

Exploiting localized heat-generation density and the resulting enhanced temperature-rise for controlled growth of carbon nanotubes (CNTs) is reported, and its potentials for batch-production of high-quality CNT probes are demonstrated. Optical near field chemical vapor deposition (ONF-CVD) benchtop fabrication schemes are developed for the localized integration of individual well-aligned carbon nanotubes without bending/buckling exactly at desired nanoscale sites. It is demonstrated that generating self-aligned catalyst nanoparticles superimposed on top of silicon nanotips, along with near-field induced absorption confinement, provide the ability to localize the generated heat at the nanotips apexes, and control the CNT growth locations. The nanoscale maskless controllability of the growth site is shown by properly tailoring ONF-CVD conditions to overcome overall heat exposure of the substrate for selective activation of catalyst nanoparticles located at apexes, from those dispersing all over the tips. The calculated local power densities and temperature profiles of the simulated tips, clearly demonstrate the confined heat and optimal gradient of generated temperature rise as the main factors affecting the growth. In addition to determining necessary processing conditions to control the localization and orientation of the growth, parameters affecting the length and diameter of the localized individually grown nanotubes are also presented. Optical near-field-based growth schemes can be extended for localized maskless fabrication of other nanoscale devices, beyond the diffraction limit, using photothermal effects.

## 1 Introduction

Over the last decade, innovative designs for the scanning near-field optical microscope (SNOM) probes have been focused on plasmonic grating patterns on the lateral side of a conic [1–4]/pyramidal [5, 6] tip, tapering the metallic waveguide/AFM tip [7, 8], or embedding a metal nanoparticle at the apex of the tip [9, 10] in the case of apertureless probes, and specific plasmonic nanostructures including bowtie [11, 12], C-shape [13], tip on aperture [14, 15], conic rings [16], an extraordinary nanohole [17], and a slit with a groove array [18] on top of the aperture in the case of apertured SNOM probes. These nanoantenna probes based on the noble metal nanostructures have been exploited to achieve efficient throughput and adiabatic nanofocusing of light, as well as enhanced polarizabilities at optical wavelengths arising from the excitation of the localized surface plasmon resonances (LSPRs) [19, 20]. These features can be utilized in diverse applications including tip-enhanced Raman/photoluminescence spectroscopy [21, 22], fluorescence-based sensing [23], near-field microscopy [24, 25], optical tweezers [26], and nanocolor sorting [27, 28].

Vibration isolation, tip-sample spacing control feedback, and detection optics are some of the issues that have already been considered in most SNOM system designs. A critical limitation in employing SNOM systems for the enhanced imaging and spectroscopy applications is unwanted tip-sample couplings; for instance, unwanted redshift in the spectral response of the probe, occurring through the characterization of plasmonic/metallic specimens [28], due to the dependency of the LSPR features on the optical properties of the specimen material [19]. Several designs have been reported to mitigate this problem in the case of apertured SNOM probes, including mapping nanoantenna modes in simultaneously optical near-field phase and amplitude distributions using phase-sensitive heterodyne interferometry [29], mapping the near-field magnetic distribution of the plasmonic nanoantennas with a hollow pyramidal probe [30], and controlling carrier concentration via electrostatic gating of graphene coating on a bowtie aperture of the SNOM probe [28]. In the case of apertureless SNOM probes, even though methods based on modifying the detection approach as in photo-induced force microscopy (PiFM) [31–33], and the interferometric phase mapping [34] have been reported, it is shown that carbon nanotube (CNT)-based scattering SNOM probes are ideal candidates to resolve the mentioned unwanted couplings [35, 36]. Mapping plasmon-enhanced and extremely strong optical fields (e.g., in the case of nanoscale pinholes, deep gratings, and hard-to-access gaps between plasmon-resonant nanoparticles) can be facilitated using these probes [35, 36]. This is due to the fact that the polarizability, and hence the dipolar moment of the weakly interacting CNT-probe is much smaller than that of the plasmonic sample, negligibly distorting the strong scattering field produced by the sample [35].

On the other hand, the fast development of the novel three-dimensional (3D) nanoelectronic and nanophotonic devices, e.g., photonic crystals with subwavelength structures [37], demands atomic force microscopy (AFM) technique capable of high-resolution imaging of high-aspect-ratio topographies, containing deep/narrow vias/trenches. However, the conventional silicon (Si) AFM tips are brittle, and have a limited lifetime, due to the intrinsic mechanical/chemical instability of Si. The low wear resistance of the tip material (Si) leads to significant degradation of the spatial resolution in long-term scanning [38]. Furthermore, the geometry of silicon pyramid/cone tips usually limits tracing sharp topographic variations of the specimen, especially for the high-aspect-ratio critical-dimension surface features. Hence, there has been a growing demand for AFM probes having not only a small radius of curvature but also a high aspect ratio (>10:1) for imaging with high lateral resolution. Complicated nanofabrication procedures, including focused ion beam (FIB) [39] or focused electron beam (FEB) [39, 40] processes, which have been utilized for the production of commercially accessible probes with relatively high aspect ratio, cost at least 10 times higher than the fabrication methods for conventional AFM probes. Furthermore, the high density of defects entered via FIB/FEB process makes the tips fragile and susceptible to breakage. In this regard, CNT-based AFM probes are also potential candidates for remarkably promoting the image resolution and probe lifetime, with no need for complicated nanofabrication processes. The resolution enhancement is due to the inherent nanometric diameter and the high aspect ratio of CNT to fully map the bottom of vias/trenches without generating artifacts [41–44]. The CNT-probe lifetime results from outstanding mechanical robustness and bending flexibility of the CNT hexagonal graphitic lattice to elastically sustain structural integrity during large axial deformations [44].

Some recent studies have been devoted to the controlled growth and alignment of CNTs to enable new functionalities for high-end applications, such as next-generation electronics. Several examples are including chirality controllable synthesis to realize large-scale fabrication of single-wall carbon nanotubes (SWCNTs) with a specific chiral index [45], aligned nanotubes assembly with covalent atomic bridges to enhance unidirectional electronic transport [46], spontaneous global alignment of CNTs for preserving one-dimensional quantum properties of nanotubes on a macroscopic scale [47, 48], and self-organization of few-wall CNT coils for the realization of miniature devices based on electromagnetic coils, such as inductors [49]. Meanwhile, a controlled growth scheme for the localized integration of an individual free-standing well-aligned carbon nanotube, without bending/buckling precisely at the desired nanoscale site (e.g., tip apex), remains challenging. To produce CNT SNOM/AFM probes, various methods have been developed. Adhesive CNT-attachments using an optical microscope and micromanipulators [41, 42], applying an electric field [43, 44, 50, 51], arc discharge [51, 52], or electron beam [53] between the tip and a prefabricated nanotube cartridge source are some of the early examples. The connection point of the nanotube to the tip apex in these lengthy methods can be hardly adjusted even using a scanning electron microscope (SEM), and a nanotube bundle can be attached to the apex. Solution-based assembly methods such as dielectrophoresis (DEP) [54–56] and solvent evaporation [56], as well as the magnetic- [57] and microwave- [58] assisted methods, have drawbacks of low controllability of the process over the alignment, curvature, and length of the attached nanotube or bundle. Chemical vapor deposition (CVD) growth methods, along with picking up a droplet of catalyst solution by adjusting a trigger threshold to deflect the cantilever beam [59], generating nanopores on top of a flattened silicon pyramid followed by catalyst deposition [60, 61], or catalyst coating all over the tip and using surface guiding effect [62] have also been reported. In spite of the increased bonding strength of the grown nanotube to the tip, such fabrication procedures usually have a low success rate and reproducibility, due to the difficulty in placing a catalyst nanoparticle at the desired site, or in generating not-looped individual nanotubes. Some other fabrication processes with the aim of increasing CNT-tips throughput have been attempted [63–68]. Nonetheless, the inherent global heating of the specimen in these thermal-CVD-based processes leads to the low usable probe yield, as a result of the natural curvature and random orientation of the resulting, not free-standing, thermal-CVD CNTs [66], or due to the little control of the thermal process over density [63, 65], site [65, 67], and length [68] of the grown CNTs. Additional post-processing techniques including straightening [66], or shortening [67] the CNT are thus necessary.

In this paper, to solve the above-mentioned problems, through the development of optical near field (ONF) based growth schemes, we show how a large gradient of generated temperature rise can be exploited for the controlled growth of carbon nanotubes. It is demonstrated that the designed optical near field chemical vapor deposition (ONF-CVD) technique, applied to the silicon nanotips having self-aligned catalyst nanoparticles on top, can result in nanoscale confinement of the generated heat, and consequently localized growth of individual well-aligned nanotubes at desired locations, i.e., apexes of silicon nanotips. The maskless selective controllability of the ONF-CVD process in nanoscale is proved through the fact that even though catalyst nanoparticles are spread all over the sidewalls and apexes of a silicon tip array, the CNTs are grown only at the apexes. This is realized by overcoming the overall heat exposure of the substrate to generate a confined heat density at the tips apexes due to the near-field nature of the process (i.e., not limited by diffraction), as well as proper adjustment of the irradiation laser power to generate a large enough temperature-rise only at the tips apexes required for growing nanotubes. The resulting temperature profiles from calculated absorption power densities for the simulated tips clearly demonstrate the significant role of the near-field enhancement effects for the CNT growth in the performed processes. Along with controlling the desired growth location and alignment, the capabilities of the proposed methodology to control the length and diameter of the individual nanotubes are also presented.

## 2 Results and discussion

**Figure 1:**
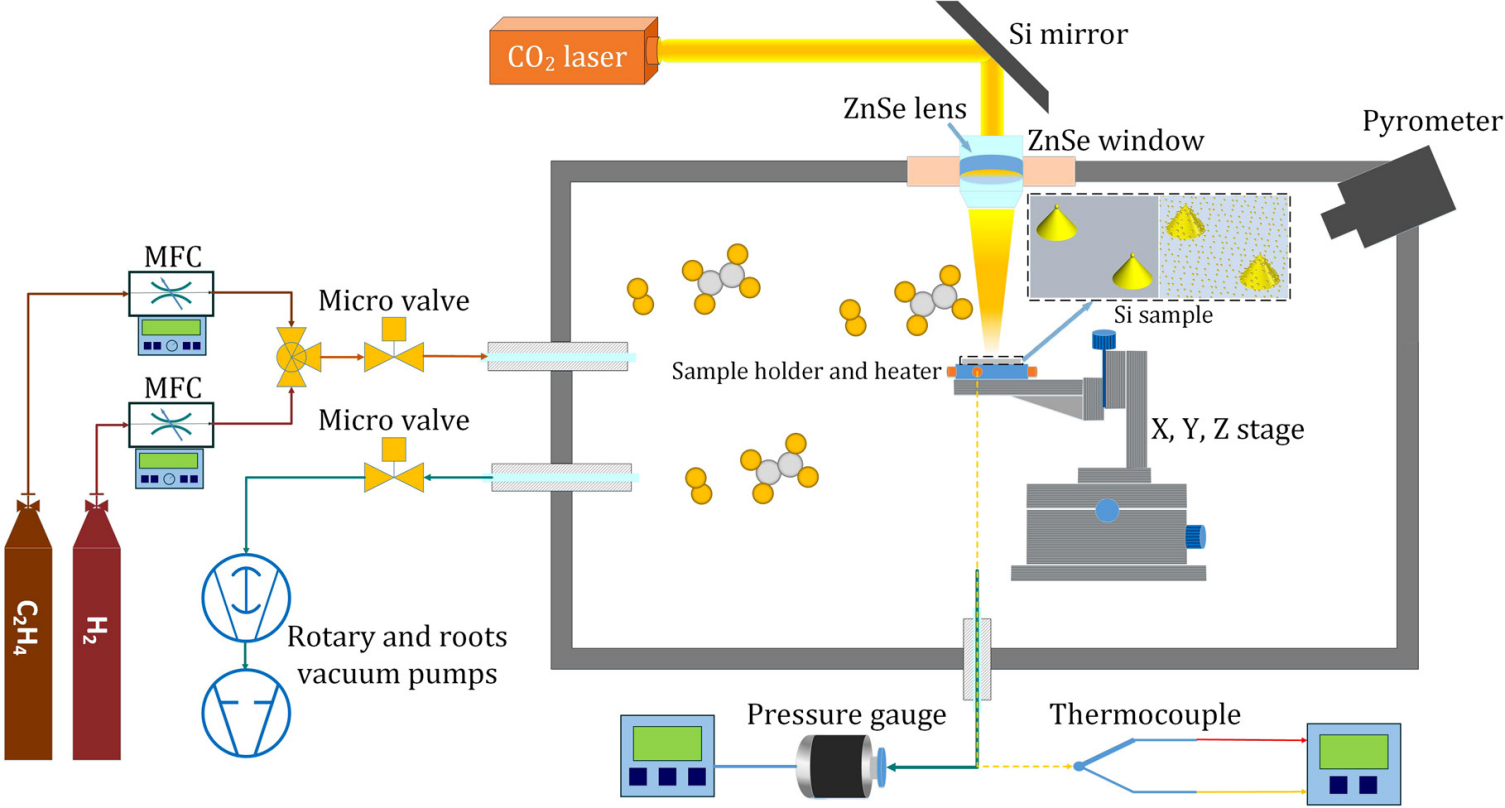
Schematic illustration of the experimental setup for ONF-CVD growth of carbon nanotubes. The Si sample is containing silicon nanotips along with catalyst nanoparticles at the apexes or apexes and sidewalls of the tips.

In this section, ONF-CVD setup and growth schemes designed for localized integration of individual well-aligned carbon nanotubes at desired nanoscale sites are explained. The experimental setup for optical near field CVD process is shown schematically in Figure 1. As the substrate, an n-type Si wafer with <100> crystal orientation, having 1–5 Ω cm resistivity is used, on which silicon nanotips with catalyst nanoparticles at the apexes or apexes and sidewalls of the tips are then fabricated. In the center of a cylindrical stainless steel chamber, a substrate holder equipped with a micro-positioner (X–Y–Z stage) is located (see Figure 1), which can be heated resistively (up to 150 °C), apart from radiant heating of the IR laser tube. The beam of a continuous wave (CW) CO_2_ laser hits the sample after reflecting from a 45° tilted Si mirror, and passing through a zinc selenide (ZnSe) window (see Figure 1). The laser spot on the specimen is about 2 mm in diameter, which can be reduced to 100 μm using a ZnSe lens in focus. The laser wavelength is tuned to 10.532 μm for the CNT growth in the ethylene (C_2_H_4_)/hydrogen (H_2_) medium, due to the fact that resonant vibrational excitation of C_2_H_4_ molecules (i.e., *ν*_7_ absorption band or CH_2_ wagging mode of the ethylene molecules) coincides at this wavelength. Hence, not only efficient laser energy coupling into the growth reactions (i.e., enhanced growth) is possible [69–71], but also photo-dissociation of the precursor gas can be performed via the gas phase heating in such a cold wall reactor system, in addition to the adsorption-phase pyrolytic processes on the substrate [72, 73]. The temperature inside the laser spot is measured optically using a pyrometer, while the average temperature of the substrate can be measured with a K-type thermocouple, connecting directly to the sample holder, in contact with the substrate. High purity ethylene and hydrogen gases can be entered into the chamber, using mass flow controllers (MFCs), and electrically controlled micro-valves. The initial vacuum (resulted from rotary and roots pumps), and the operating pressure are measured using pressure gauges.

### 2.1 Fabrication of CNT-tips based on applying ONF-CVD process to tips having self-aligned catalyst particles

The fabrication steps of the designed scheme for the CNT growth on tips, based on performing the ONF-CVD process using a silicon tip array, having self-aligned catalyst nanoparticles on top, are described in the following.

#### 2.1.1 Generation of catalyst nanoparticles on silicon substrate

**Figure 2:**
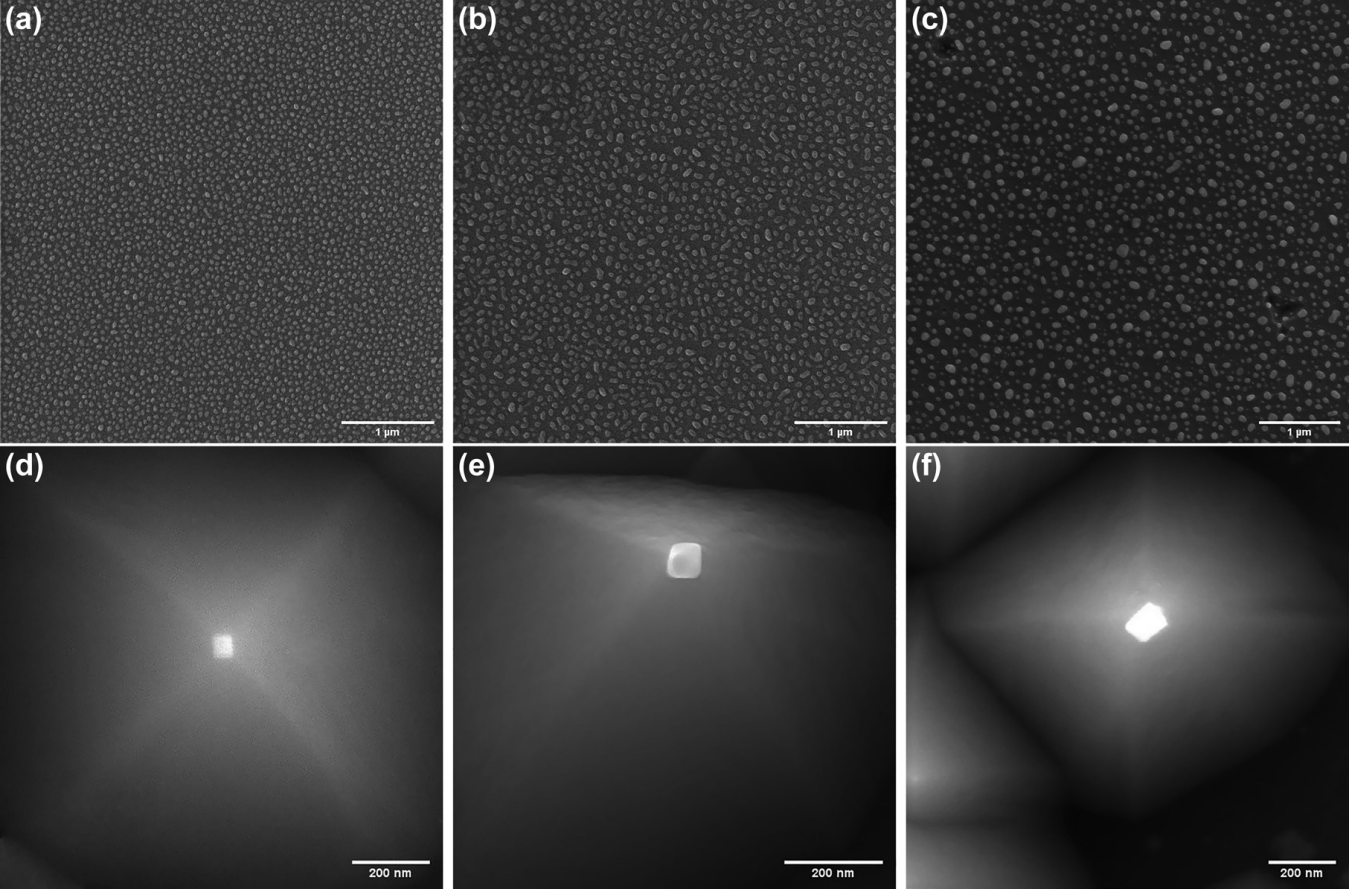
Catalyst nanoparticles and their typical underlying silicon nanotips generated for the three initial catalyst depositions. 5 nm (a, d), 10 nm (b, e), and 15 nm (c, f) thick deposited Ni-layer, 2 min IR irradiation with the power of 30 W (a), 40 W (b), and 50 W (c), and anisotropic etching in a 20% KOH solution at 65 °C for 3 min (d), 4 min (e), and 5 min (f) are used.

Catalyst nanoparticles are formed on the Si substrate, first by sputtering an appropriate thickness of nickel (Ni), and then exposing and heating the deposited layer using IR (10.6 μm) laser irradiation in a reducing environment of hydrogen. Figure 2a–c shows the SEM images of the catalyst nanoparticles generated from different initial Ni-layer thicknesses of 5, 10, and 15 nm, respectively, through the irradiation in the hydrogen medium. The details of the RF sputtering and heating processes can be found in the Methods section. As can be seen in Figure 2a–c, the average size of the formed catalyst particles is increased with increasing the initial thickness of the deposited catalyst layer. The average particle size for the initial catalyst thicknesses of 5, 10, and 15 nm are ∼36, ∼60, and ∼90 nm, respectively.

#### 2.1.2 Formation of silicon nanotips with self-aligned catalyst nanoparticles on top

**Figure 3:**
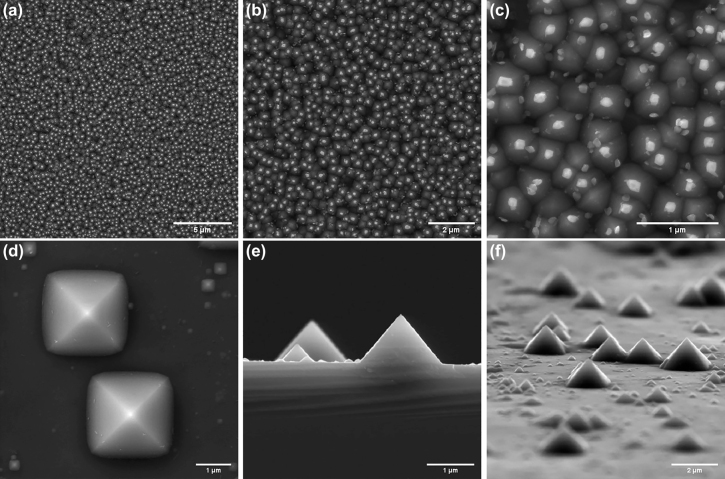
Formation of self-aligned catalyst nanoparticles on apexes, and completely fabricated nanotips. (a–c) Different magnifications of a typical being-formed array using nanoparticles masks and anisotropic etching in a 20% KOH solution at 65 °C. The thickness of the deposited Ni layer is about 15 nm. Top surface (d), cross-sectional (e), and tilted (f) SEM images of generated nanotips without catalyst particles. The etching time is about 6 min.

In the nanotips fabrication process, the generated catalyst nanoparticles are utilized as the masks for anisotropic etching of the underlying Si substrate. Through this step, the catalyst nanoparticles will exactly be self-aligned on top of the tips. Hence, the growth of carbon nanotubes can be controlled at the desired locations, i.e., tips apexes, where the nanoparticles are located. Typical generated silicon nanotips along with their superimposed Ni nanoparticles, corresponding to the three catalyst thicknesses of 5, 10, and 15 nm, are shown in Figure 2d–f, respectively. It is worth mentioning that the process of forming silicon nanotips with catalyst nanoparticles on top requires stopping the etching process at a certain time range (see the Methods section). For clarity, a typical silicon tip array being formed beneath the catalyst (Ni) nanoparticles is represented in Figure 3a–c. By continuing the etching process, first, the tips have been further sharpened and thereby the catalyst nanoparticles have been fallen, resulting in completely fabricated silicon nanotips without catalyst nanoparticles, as shown in Figure 3d–f. Then, the sharpened silicon nanotips are gradually become blunt and flattened, due to the lack of the nanoparticles masks. In this regard, it can be seen that the number of catalyst nanoparticles in Figure 3a–c is decreased compared to that in Figure 2a–c. This is mainly due to the size distribution of the catalyst nanoparticles for a given deposited catalyst layer. The resulting tips from catalyst nanoparticles with a smaller size than the average size are formed and sharpened faster, and hence their corresponding nanoparticles (which act as the masks) are fallen sooner in the KOH solution. These catalyst-free tips become flattened before forming all silicon nanotips, resulting in the fewer remained nanotips and their corresponding nanoparticles.

#### 2.1.3 Controlled ONF-CVD growth of individual well-oriented CNTs on tips apexes

**Figure 4:**
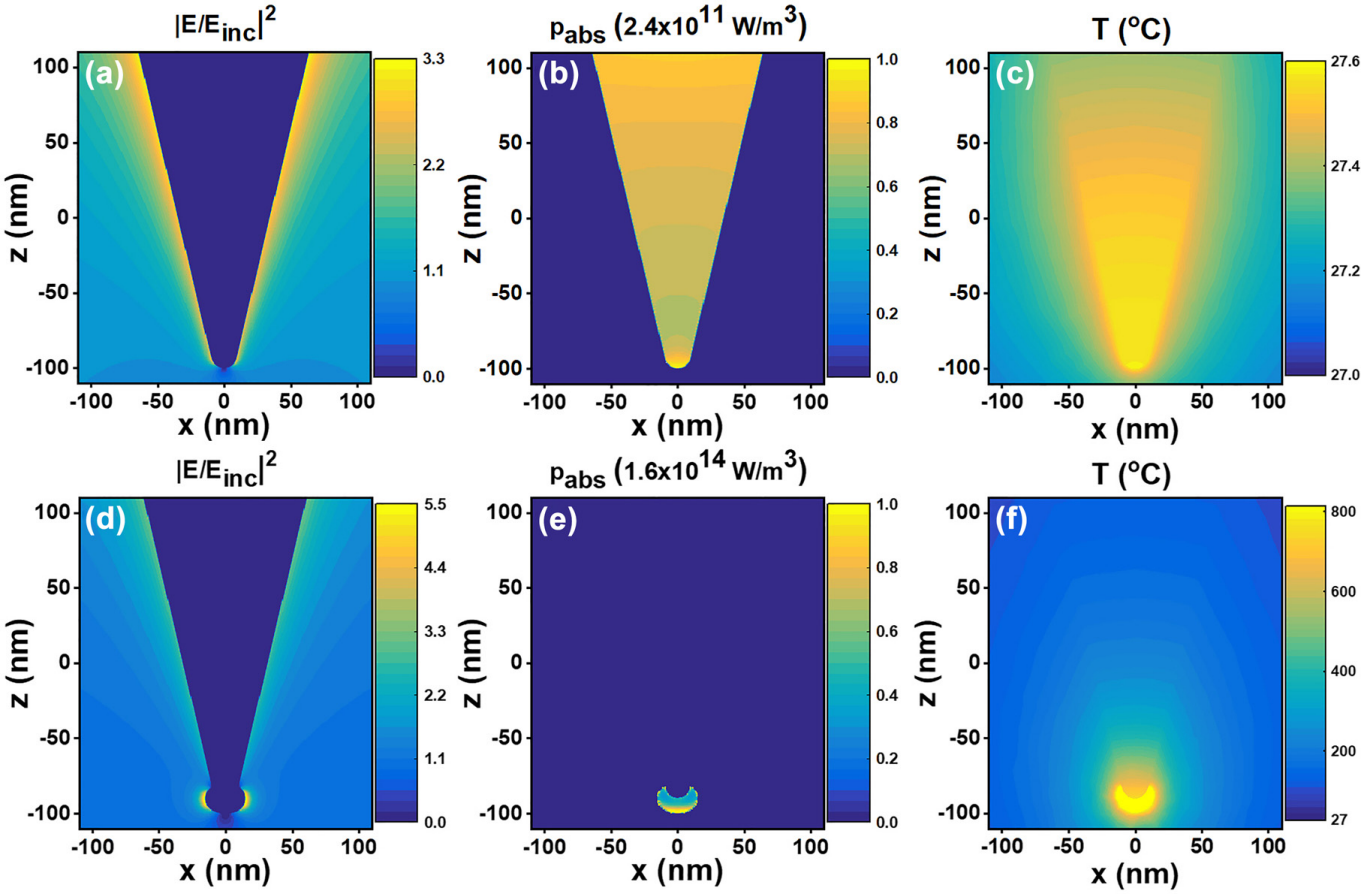
Simulation results for a silicon tip with and without a Ni nanoparticle on top. (a–c) Normalized near-field intensity, heat generation density, and temperature-rise distribution in the *xz* plane for a silicon tip. (d–f) Corresponding results for a silicon tip with an ellipsoidal Ni nanoparticle on top.

The ONF-CVD growth process has been performed utilizing the silicon nanotips topped with catalyst nanoparticles (see Figure 2d–f). In the properly performed ONF-CVD process (see Methods for the process parameters), the growth condition is adjusted by tuning the CO_2_ laser power to provide a large enough temperature rise required for growing nanotubes. The resulting insufficient temperature increase inside the laser spot for the tuned power, optically measured with the pyrometer (see Methods for the details), confirms localized heating only for the desired sites on the substrate. The simulation result in Figure 4e also shows that the generated heat can be significantly confined to the Ni nanoparticle on top of the silicon tip (see Methods for the simulation details). Furthermore, a significant enhancement in the generated absorption power density, at least two orders of magnitudes, can be observed for the silicon tip topped with Ni nanoparticle compared to that not having it (see Figure 4b and e). This enhancement (see Figure 4e) is due to the absorption of Ni nanoparticle at the illuminating wavelength, which can lead to increasing the temperature of the tip apex up to the growth temperature, as can be seen in the profile of Figure 4f. The corresponding temperature profile for the alone silicon tip indicates negligible temperature rise (Figure 4c), due to the very small extinction coefficient and thereby low absorption coefficient of Si at the IR wavelength of 10.532 μm [74]. The calculated local electric field intensity (normalized to the incident field intensity) for the tip having Ni nanoparticle on top shows an enhancement at the apex, which is about 5 times larger than that at the rest of the structure (see Figure 4d), while the corresponding profile for the alone silicon tip (Figure 4a) shows no considerable enhancement at the apex, due to the Si optical properties at 10.532 μm. The enhanced field intensity at the tip apex along with the much larger absorption coefficient of Ni compared to Si result in the enhancement of the local heating and thereby much higher temperature rise at the apex of the tip with Ni nanoparticle (Figure 4f) compared to that without it (Figure 4c).

The resistive heater can be also used for initial biasing of the substrate temperature, e.g., 150 °C, measured by the connected thermocouple. This initial temperature-biasing leads to the increasing temperature-rise profile by an offset relative to the room temperature. Moreover, it can lead to the absorption enhancement, thus more temperature-rise of the silicon substrate by laser irradiation. It has been shown that for the n-type silicon, transmission at the temperature of 150–500 °C is decreased approximately 4.4–64% than that at the room temperature, which leads to an enhancement in the absorption coefficient by a factor of 2.1–30 [74], and correspondingly the same increase in the temperature rise. Hence, the required thermal energy for growing CNT on the tip apex can be reached at a lower irradiation power.

**Figure 5:**
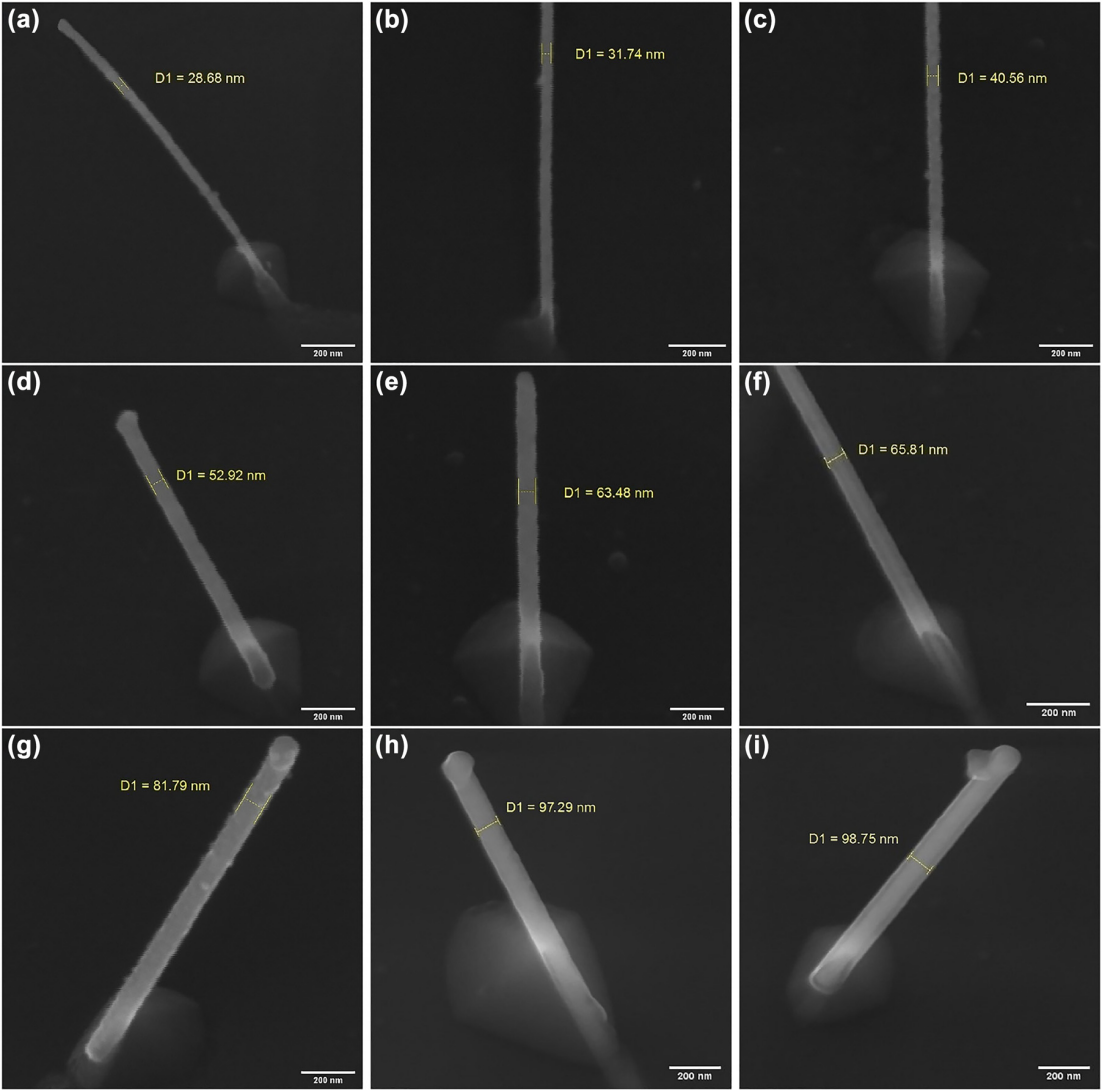
Localized ONF-CVD fabrication of CNT-tips. Typical examples of the grown individual free-standing well-oriented carbon nanotubes without bending/buckling at the tips apexes for the case of 5 nm (a–c), 10 nm (d–f), and 15 nm (g–i) initial catalyst deposition. The approximately measured diameter of each nanotube is shown in yellow.

Figure 5 illustrates the SEM images of the fabricated CNT tips corresponding to the three initial catalyst depositions, by applying the ONF-CVD process to the nanotips having self-aligned Ni nanoparticles. As can be seen, the individual free-standing well-oriented carbon nanotubes, with a high aspect ratio of approximately 10:1 − 30:1, and a protruding length of around 1 μm, have been grown at the desired sites (i.e., tips apexes). Based on the SEM images, the diameter distributions of the grown nanotubes on the tips apexes can be divided into three categories of 28–45, 50–68, and 80–100 nm, which the corresponding examples are shown in Figure 5a–i respectively, for the three initial catalyst thicknesses of 5, 10, and 15 nm, respectively. It can be observed that the nanotubes are straight, without bending/buckling along their lengths, which has been ascribed to the decreased defect density [69].

**Figure 6:**
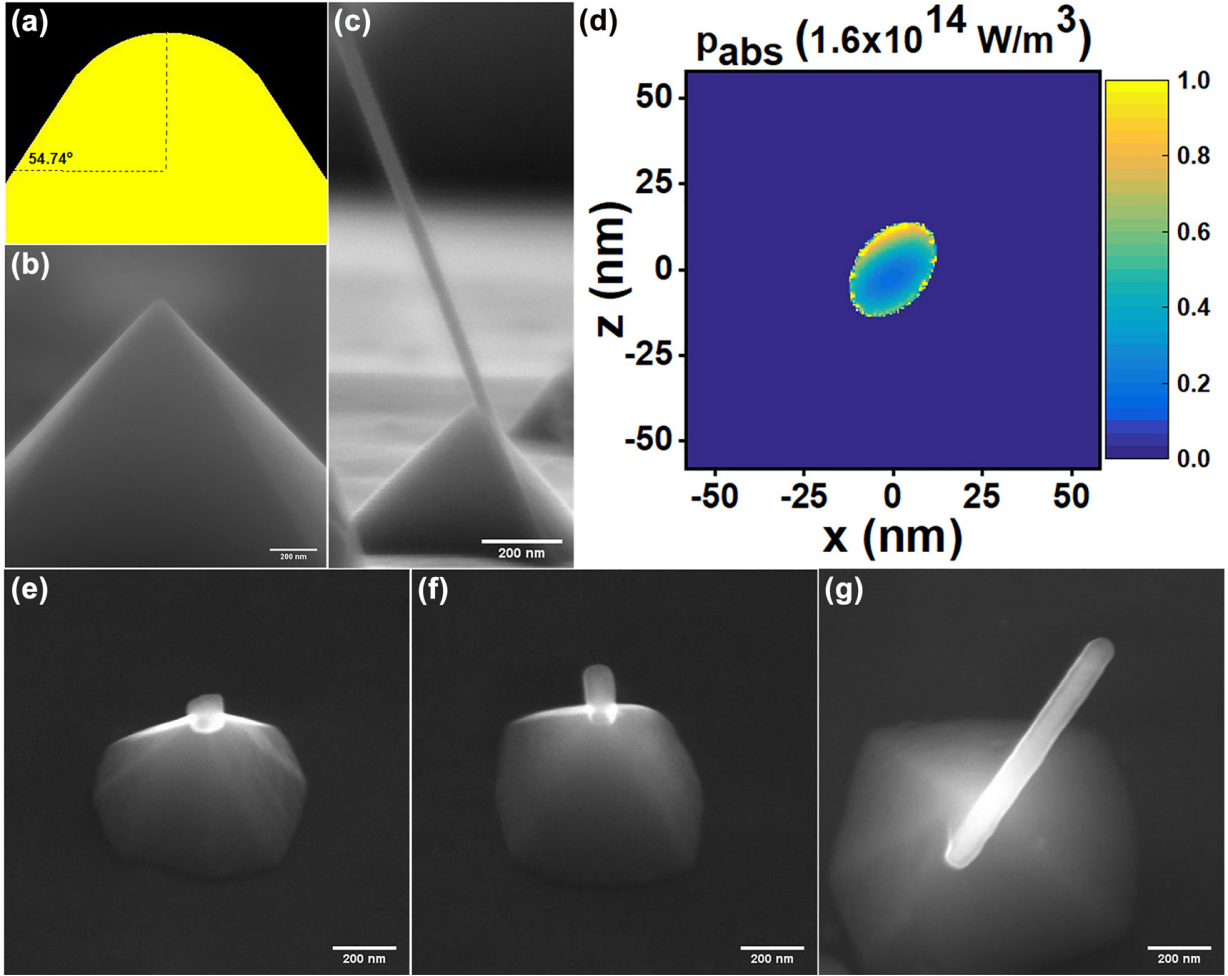
The effect of apex curvature and irradiation time on the grown nanotube of the CNT-tips. (a) Schematic of a tip apex, indicating the apex curvature and the slope of the lateral edge of the tip. (b) SEM image of a fabricated tip, indicating apex curvature with a diameter of about 36 nm. (c) Cross-sectional SEM image of a typical CNT-tip, including the grown CNT along the lateral edge of the pyramidal tip with the angle of 35.26° to the normal. (d) Heat generation density for an ellipsoidal Ni nanoparticle with a maximum tilt angle of 54.74° to the surface. The tilted profile inclines the growth of nanotube along the lateral edge of the tip. (e–g) CNT-tips for which the nanotubes are grown at different irradiation times of 15 s (e), 30 s (f), and 2.5 min (g) in the ONF-CVD process.

According to the resulted CNT-tips, the grown nanotubes are aligned vertically with an angle of about 35.26° to the normal. Due to the inherent characteristics of anisotropic V-etch of <100>-oriented Si, the sidewalls of the pyramids are formed at a slope of 54.74° relative to the surface (as in Figure 6a and b), forming an apex angle of 70.52° (2*θ*). Hence, the nanotube is inclined to be oriented with the angle of tilted nanoparticle sitting on the apex, resulting from tip sharpening with a radius of curvature, being about 35.26° or less to the normal. A cross-sectional SEM image of a typical CNT-tip, having the grown nanotube with an angle of 35.26° on the lateral edge of the tip, is shown in Figure 6c. This kind of orientation results in the particularly useful configuration of the CNT-tips for probing bottom edges of very narrow features [75]. As shown in Figure 6d, for an ellipsoidal Ni nanoparticle with a maximum tilt angle of 54.74° to the surface, the profile of heat generation density is also tilted such that the growth of nanotube can occur along the lateral edge of the tip. For simplicity, the existence of the silicon tip is not considered, due to the very small absorption of Si at the illuminating wavelength, as shown in Figure 4b and e.

It should be noted that decreasing/increasing the laser irradiation time can result in the decrease/increase of the length of the grown nanotube at the tip apex. Figure 6e–g shows three typical samples of CNT-tips whose nanotubes have been grown at different irradiation times of 15 s, 30 s, and 2.5 min, respectively. As reported [76, 77], the nanotube yield (in our case being the ultimate length of individual CNTs grown on top of the tips) would be limited as a result of decreasing the initial growth rate at the individual nanotube level (or decreasing the active catalyst nanoparticles). In this regard, the proposed ONF-CVD process can be well controlled by adjusting the incident laser power and exposure time. In addition, this process has the potential of providing dynamic information about individually growing nanotubes from distinct activated catalyst nanoparticles, by exploiting additional real-time optical characterization methods, like *in-situ* Raman monitoring [78, 79].

#### 2.1.4 Proving CNT nature of the grown nanostructures on top of the tips

**Figure 7:**
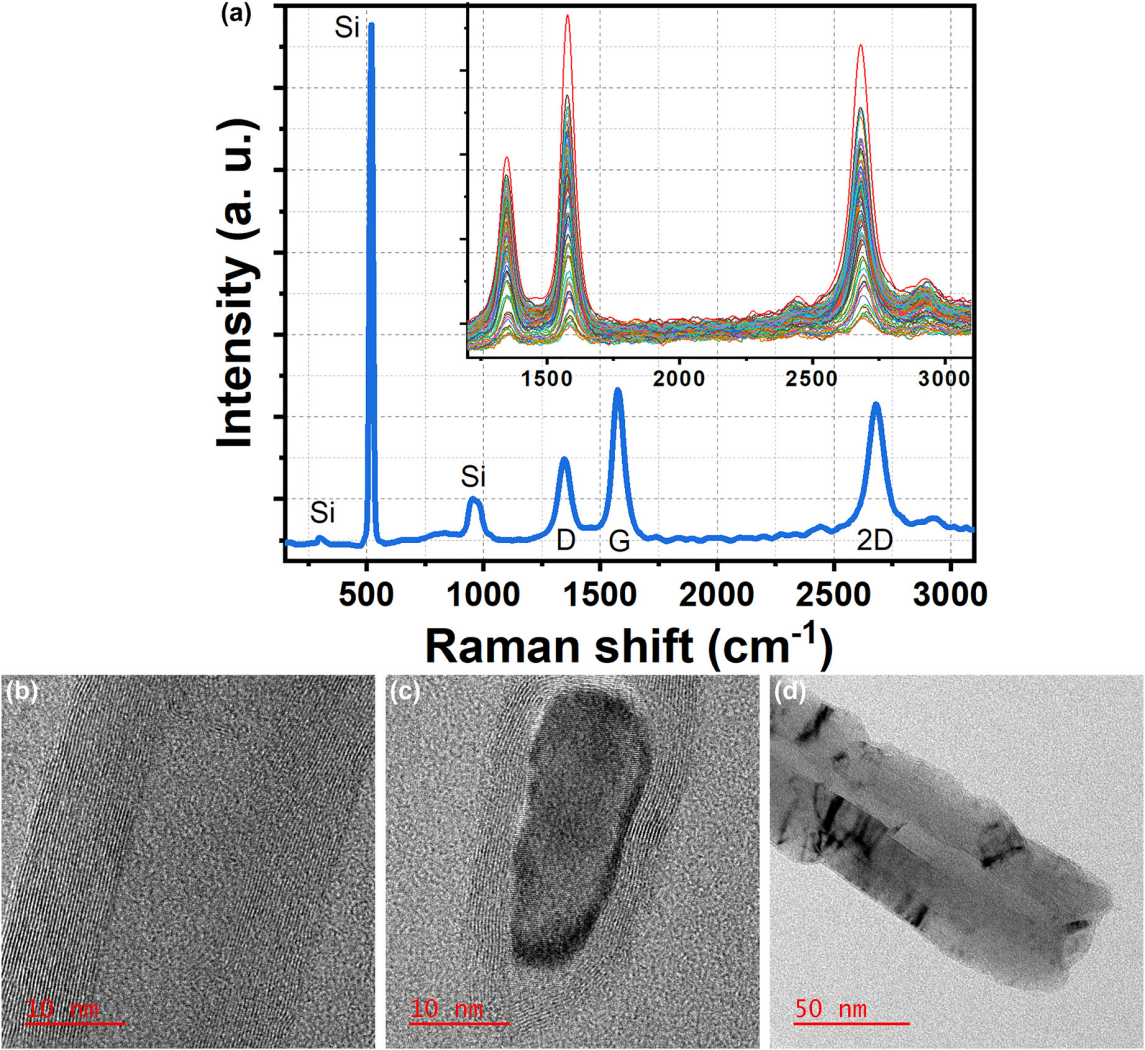
Characterizations of the ONF-CVD grown nanostructures. (a) A typical Raman spectrum of a sample including the grown nanostructures on top of the silicon tips. The inset shows the D-G regions of the resulted Raman spectra for the same sample, from an area of approximately 100 × 100 μm^2^. The spectra are clearly demonstrating the characteristics of multi-wall carbon nanotubes. The Raman excitation wavelength is 532 nm. (b) High-resolution TEM image of the outer walls, containing 29 graphitic sheets, and the channel of a multi-wall carbon nanotube grown by the ONF-CVD process. (c) High-magnification image of a nanotube tip indicating encapsulated catalyst (Ni) nanoparticle, and the well-graphitized concentric sheets. (d) TEM image of a nanotube tip with the diameter of about 60 nm, revealing the hollow structure of the nanotube without the inclusion of the catalyst nanoparticle.

Raman analysis can be used to prove the CNT nature of the grown nanostructures on top of the silicon tips, considering the fact that suspended nanotubes have a strong Raman signal [80]. Figure 7a shows the Raman spectrum of a typical sample consisting of the nanostructures grown on top of the silicon tips. This spectrum reveals the three characteristic peaks of multi-wall carbon nanotubes (MWCNTs); the D-band, G-band, and G′-band (2D-band) peaks are observed at the frequencies of 1348, 1574, and 2696 cm^−1^, respectively. The G-band, a characteristic of graphite, is representative of the graphitic structures of CNTs, whereas the D-band results from *sp* ^2^ disorders and defects (e.g., vacancies and substitutional hetero-atoms) in the graphite structures of carbon materials [81]. The G′-band (at 2696 cm^−1^ in Figure 7a), relating to the D-band with frequency combination (2 × 1348 cm^−1^) [82], is an inherent Raman property for the nanotubes, which appears even when the D-band is thoroughly absent for the defect-free ones [83]. The relatively large G/D intensity ratio in the spectrum of Figure 7a indicates the high quality of the corresponding nanotube on top of the tip. The absence of low-energy radial breathing mode (RBM), the feature specific to the single-wall carbon nanotubes, confirms the multi-walled nature [84] of the grown nanotube. The large and sharp peak in 520 cm^−1^, as well as the peaks in 300 and 954 cm^−1^, are all related to the silicon nature [78, 84] of the substrate, and fabricated Si tips.

Moreover, an area of approximately 100 × 100 μm^2^ from the sample is selected, and the D-G regions of the resulted Raman spectra (with a constant spatial spacing in the *x*− and *y*− directions) are shown in the inset of Figure 7a. Although the D-band peak in the resulted Raman spectra is observed around 1350 cm^−1^, the peak position of G-band is down-shifted from 1584 to 1574 cm^−1^, due to the higher growth temperature [77–79] near the center of the laser spot; higher growth temperature results in higher CNT graphitization level and wall quality [84], due to softening the C–C bonds [77]. In addition, for the lower spectrum (in orange) in Figure 7a inset, compared to the upper one (in red), it can be seen that the D and G peaks are broader (D-band and G-band full width at half maximum (FWHM) of 94.71 and 69.67 cm^−1^, respectively, compared to 68.84 and 59.66 cm^−1^, respectively), and the G/D intensity ratio is smaller (1.11 compared to 1.56). Hence, the corresponding grown nanotube is more defective [85]. The nanotube yield is also low, in accordance with the low intensity of the G-band peak [76]. For the upper spectrum, the FWHM of the G peak and the relative intensity of the D peak are decreased, due to the lower amounts of amorphous carbon present at the higher temperatures by increasing the reactivity of hydrogen [85]. The peak around 2930 cm^−1^ is the sum of the D-band (∼1350 cm^−1^) and G-band (∼1580 cm^−1^) peak frequencies (see spectra in Figure 7a inset). The other second order Raman peak being observed around 2443 cm^−1^ has been reported previously for the CVD grown multi-wall carbon nanotubes [82].

The produced MWCNT samples are further characterized by HRTEM analysis. The resulted images shown in Figure 7b–d reveal the details of the hollow structure of the nanotubes. The 29  well-graphitized concentric graphitic sheets distinguished around the tube channel of the grown nanotube shown in Figure 7b verify the multi-walled nature of the structure. The nanotube tips with and without the inclusion of the catalyst nanoparticle by the carbon tube can be observed in Figure 7c and d, respectively. The lattice fringes in the sidewalls of Figure 7b and c, corresponding to the interlayer distance of the (002) graphene planes, show the lack of attached non-tubular byproducts like amorphous carbon.

### 2.2 Fabrication of CNT-tips merely based on localized heat generation induced in sharp tips apexes

**Figure 8:**
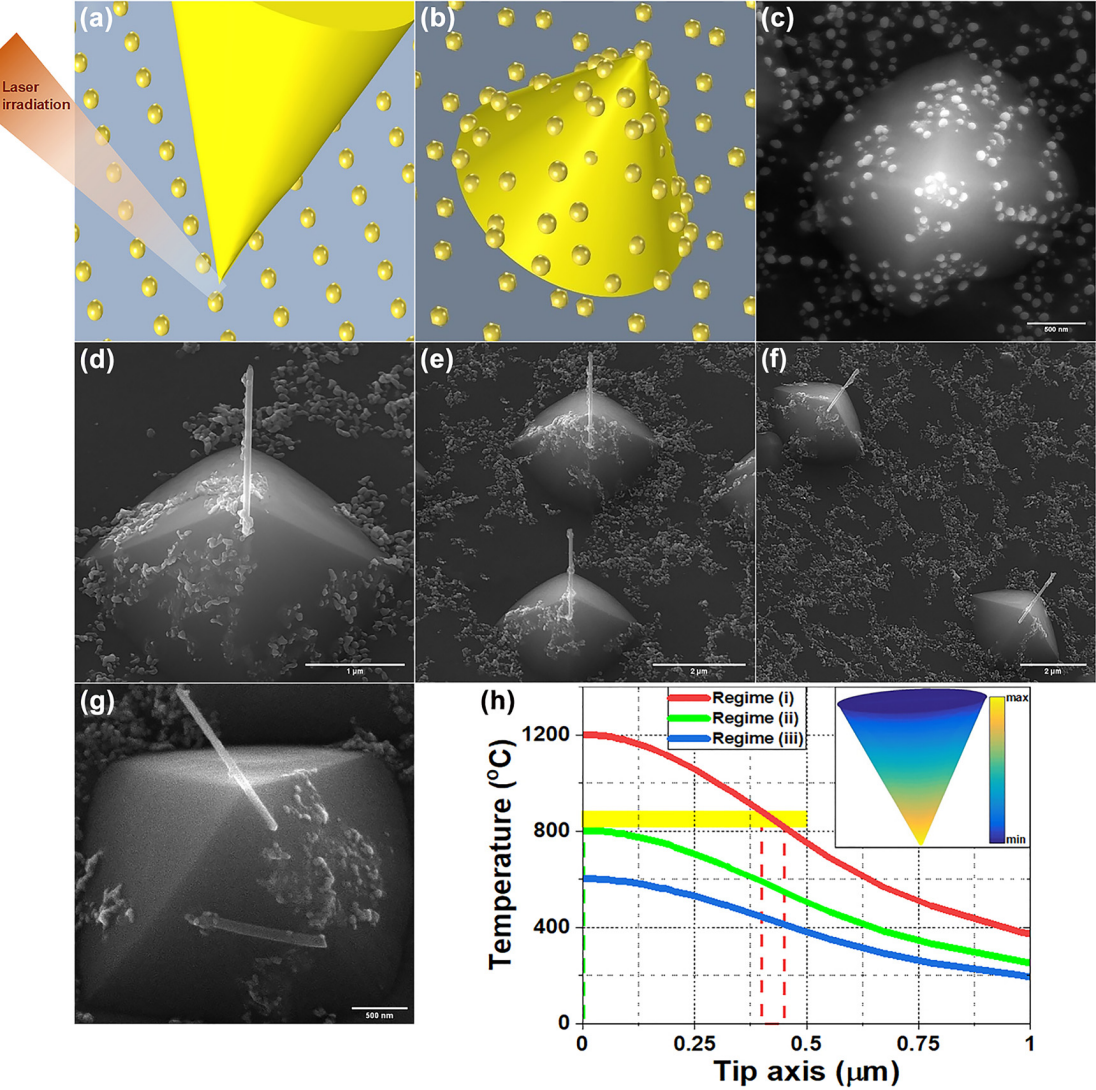
Controllability of the localized growth in the second designed ONF-CVD process. (a, b) Schematics to demonstrate conditions for the activation of only one catalyst nanoparticle as a result of the near-field induced heat generation, either locating just below the tip (a), or locating on the apex of a nanotip (b). (c) Corresponding to the schematic indicated in (b), SEM image of a fabricated silicon nanotip (in an array) containing dispersed Ni nanoparticles all over the apex and sidewalls of the tip as well as the substrate surface. (d–f) Controlled growth of individual well-aligned nanotubes for the fabrication of CNT-tips based on applying ONF-CVD process to the nanotip array with dispersed catalyst nanoparticles all over. (g) SEM image of the undesired regime (i), mentioned in the context, for an applying laser power of 70 W, showing the role of irradiation laser power in the performed ONF-CVD process. (h) The resulting temperature profiles along the axis of a simulated Si tip with 10 nm Ni cladding, indicating the three mentioned regimes in the experiments. The yellow strip shows the temperature range desired for the CNT growth on the tip. The dashed green and red lines show the regions of the tip in regimes (ii) and (i), respectively, where the nanotube can be grown. The inset shows a typical 3D temperature profile of the tip.

In order to demonstrate the localized near-field induced heat generation, and thereby confined temperature rise, as the dominant CNTs growth factor in the ONF-CVD process, providing the nanoscale controllability on the growth site, it can be shown that even if the catalyst nanoparticles are spread over the entire surface of the substrate, it is possible to control the CNT growth localized for only the nanoparticle located beneath the tip apex, where the near-field induced temperature rise could be large enough (Figure 8a). Equivalently, it can be shown that if the catalyst nanoparticles are spread all over a specimen containing nanotip (Figure 8b), only the nanoparticle located on top of the tip can contribute to the CNT growth. Hence, in this section, selective activation of the apexes catalyst nanoparticles is shown through proper tailoring of the ONF-CVD conditions.

Starting with a sample having prefabricated silicon nanotips (see Figure 3d–f), a catalyst layer is deposited, and converted into catalyst nanoparticles (see Methods for the details). The result is shown in the SEM image of Figure 8c, in which the catalyst nanoparticles are formed not only on the entire substrate surface and the sidewalls of the prefabricated silicon nanotip but also on top of the tip apex. The process of forming nanoparticles all over the tips is done just before the ONF-CVD process of CNT growth, without removing the sample from the chamber. Typically, if such a sample (as in Figure 8c) is subjected to a conventional thermal CVD process for CNT growth, CNTs are expected to be grown at all points of the sample where the catalyst nanoparticles are present [63], due to the global heating of the specimen. Here, the importance of the ONF-CVD method becomes apparent. As a result of the enhanced near-field induced absorption power density at the apexes, a high temperature rise gradient is generated along the tips, providing the ability to localized CNT growth at the desired nanoscale sites. The result of this maskless ONF-CVD process is shown in the SEM images of Figure 8d–f. As can be seen, the individual well-oriented nanotubes have been grown only on the apexes of distinct silicon tips, indicating the merely enhanced near-field induced heat generation at the apexes, as the growth factor (regardless of the existence of only one catalyst particle, as for the previous ONF-CVD process). In the properly performed ONF-CVD process (see Methods for the growth process parameters), the optimal CO_2_ laser power is fine-tuned to provide a large enough localized temperature rise only at the tips apexes required for growing nanotubes. In this way, although the catalyst nanoparticles are located all over the sample (i.e., substrate surface, sidewalls and apexes of the tips), the nanotubes can grow only on the apexes (see Figure 8d–f). Here, similar to the previous ONF-CVD process, it is possible to control the diameter and length of the grown nanotube on the tip apex by decreasing/increasing the size of dispersed catalyst nanoparticles and the laser irradiation time, respectively.

For more clarification, depending on the irradiation laser power, three different working regimes have been observed in our experiments. (i) The laser power is so high that not only the tip apex but also the tip body reaches the temperature rise required for the CNT growth. As shown in Figure 8g, for a laser power of 70 W, a nanotube has been grown on the tip body in addition to the grown nanotube on the tip apex (along the lateral edge of the tip). (ii) The laser power is fine-tuned so that an individual CNT grows only at the tip apex, as shown in Figure 8d–f for a laser power of 54 W. As mentioned, this is due to the characteristics of an ONF-CVD process, in which only the tip apex can reach the temperature required for growing nanotube, as a result of high temperature gradient generated along the tip. (iii) The laser power is low and the thermal energy needed for the CNT growth is not provided. Hence, after the growth process, the tips are still without any grown nanotubes. As can be seen in Figure 8h, by changing the irradiation power, the three aforementioned regimes in the experiments can also be clearly observed in the resulting temperature-rise profiles along the axis of a simulated tip. A typical 3D temperature profile of the simulated tip is represented in the inset of Figure 8h. The regions of the tip where the nanotube can be grown in regimes (i) and (ii), based on the temperature range desired for the CNT growth, are also shown in Figure 8h. It can be observed that the minimal temperature rise for the growth of nanotube (i.e., lower limit of the yellow strip in Figure 8h) is provided at the tip apex (green dashed line) for the regime (ii), while in the regime (i), the desired temperature range for the CNT growth (the yellow strip) coincides with a region on the tip body (indicated with the red dashed lines).

## 3 Conclusions

Controlled optical near-field CVD growth of carbon nanotubes has been demonstrated based on the confinement of the generated heat at the desired sites. It is demonstrated that performing the ONF-CVD process with the nanotips having self-aligned catalyst nanoparticles on top leads to the localized growth of individual well-aligned nanotubes. This is verified by the calculated absorption enhancement for a conic tip with catalyst nanoparticle on top, which is at least two orders of magnitude larger than that for the tip not having it. The additional controlled ONF-CVD growth scheme, implemented for a silicon tip array containing dispersed catalyst nanoparticles all over the tips sidewalls and apexes, clearly confirms the maskless selective growth of nanotubes, as a result of the enhanced heat generation at the apexes. This is realized by generating near-field induced confined heat along with fine-tuning the irradiation laser power to provide a temperature rise, large enough only at the apexes for growing nanotubes, which is clearly demonstrated by the resulting temperature profiles using numerical simulations corresponding to the experimental results. In addition to controlling the localization of the well-aligned nanotubes, control of the length and diameter of the individual nanotubes is also shown, through adjusting the IR-laser irradiation time, and the size of the self-aligned nanoparticle superimposed on the tip, respectively. The represented SEM images demonstrate proper implementation of the whole fabrication steps in the designed procedures for the controlled growth of nanotubes. The nature of the grown multi-wall CNTs is proved by the Raman and HRTEM analyses. Optical near-field-based methods have potential applications for the localized maskless fabrication of novel nanostructures, beyond the diffraction limit, using photothermal effects.

## 4 Methods

### 4.1 Sputtering of the catalyst layer

Considering the high melting temperature of Ni (1455 °C), RF-sputtering was used for depositing 5, 10, and 15 nm of Ni, with deposition times of 10, 20, and 30 s, respectively. The deposition process was performed at an RF power of 40 W, a chamber pressure of 4 × 10^−2^ mbar, and a substrate temperature of 100 °C.

### 4.2 Formation of the catalyst nanoparticles on silicon substrate

To form catalyst nanoparticles with different size distributions, three samples with Ni-layer thicknesses of 5, 10, and 15 nm were annealed at 480–600 °C by an IR (10.6 μm) heating laser with the power of 30, 40, and 50 W, respectively. Laser exposure time of 120 s in a hydrogen medium at a pressure of 1 mbar (i.e., H_2_ flow rate of 200 sccm) was used.

### 4.3 Anisotropic etching for the fabrication of silicon nanotips with self-aligned catalyst nanoparticles on top

The anisotropic etching of the silicon substrate, using catalyst nanoparticles as masks, for the three samples with initial catalyst thicknesses of 5, 10, and 15 nm was performed in a 20% KOH solution at 65 °C, for 180 ± 10, 240 ± 15, and 300 ± 20 s, respectively. Continuing the etching process resulted in further tips sharpening and consequently falling of the catalyst nanoparticles.

### 4.4 ONF-CVD growth of individual CNTs on tips with self-aligned catalyst nanoparticles on top

In the ONF-CVD process performed using silicon nanotips topped with catalyst particles, the initial pressure, H_2_ and C_2_H_4_ flow rates, and operating pressure were 4 × 10^−3^ mbar, 200 sccm, 100 sccm, and 1.5 mbar, respectively. The flow of the mixed entrance gas was laminar over the substrate. The temperature inside the laser spot, resulting from IR-laser power of 74–78 W, reached values between 360 and 400 °C, optically measured with the pyrometer. These insufficient temperatures for the CNT growth were indicating localized heating only for the desired sites on the substrate to enhance the temperature up to the CNT growth temperatures. In the case of using the resistive heater, biasing at the temperature of 150 °C, the mentioned laser power can be reduced by about 12 W. The irradiation time for the performed production runs was 150–240 s.

### 4.5 Calculation of the heat generation densities and temperature rise profiles

Three-dimensional finite difference time domain (3D FDTD) analysis method was utilized to numerically evaluate the effect of optical near field on the photothermal heating of a conical silicon tip, with or without a Ni nanoparticle on top. The height, cone angle, and radius of curvature of the tip were considered 1.8 μm, 30°, and 10 nm, respectively. The Ni nanoparticle was assumed to be an ellipsoid with 30, 30, and 20 nm in diameter in the *x*− , *y*− , and *z*− directions, respectively. The refractive index of the background medium was set to be 1. The structures along the *z*− axis were assumed to be illuminated with an unpolarized plane wave (at the wavelength of 10.532 μm) propagating in the *z*− direction toward the tip apex. The incident light intensity was assumed to be 8 mW/μm^2^. The calculated local power densities (i.e., heat generation densities) resulting from optical simulations were exploited as the sources of heat to compute the steady-state temperature profiles of the structures. For this means, heat transfer equation in the steady-state regime was solved. Thermal conductivities of silicon and nickel were considered 148, and 90 W/mK, respectively. The convective heat transfer coefficient of 10 W/m^2^K was used at the interfaces of Si, as well as Ni with the surrounding environment, and the thermal boundary conductance at the Ni–Si interface was assumed to be 100 MW/m^2^ K. The same calculations were done for achieving temperature rise profiles for the case of simulated silicon tip with 10 nm Ni cladding.

### 4.6 Formation of the completely fabricated (i.e., catalyst-free) silicon nanotips

By continuing the etching process of fabricating silicon tips beneath the catalyst nanoparticles, the complete nanotips were fabricated in a 20% KOH solution at 65 °C. The required etching time for the three samples with initial catalyst thicknesses of 5, 10, and 15 nm was determined about 4, 5, and 6 min, respectively, for the complete fabrication of almost all included tips in each specimen.

### 4.7 Generation of the catalyst nanoparticles on complete silicon nanotips

To form catalyst nanoparticles on the specimen containing silicon nanotips, first, 10 nm – thick Ni layer was sputter deposited on the substrate at the RF power of 40 W, operating pressure of 4 × 10^−2^ mbar, and a substrate temperature of 100 °C, during 20 s. The catalyst layer was then converted to the nanoparticles, using the IR-laser irradiation at the power of 40 W, for 120 s in a hydrogen medium at a pressure of 1 mbar (i.e., H_2_ flow rate of 200 sccm).

### 4.8 ONF-CVD growth of individual CNTs on tips containing dispersed catalyst nanoparticles

In this ONF-CVD process, the optimal laser power of 54 W was attained such that despite the catalyst nanoparticles are dispersed all over the sample (substrate surface, sidewalls and apexes of the tips), the nanotubes were grown only on the tips apexes, as a result of the enhanced absorption power density at the apexes, and the resulting high temperature gradient along the tips. The optically measured temperature inside the laser spot, resulting from the obtained laser power, reached values around 640 °C, which provided only the temperature increase for the CNT growth at the tips apexes. The irradiation time was 150 s. The initial pressure, hydrogen and ethylene flow rate, and operating pressure were the same as previous ONF-CVD process (i.e., 4 × 10^−3^ mbar, 200 sccm, 100 sccm, and 1.5 mbar, respectively).
